# Bacteremia and endocarditis caused by a Gordonia species in a patient with a central venous catheter.

**DOI:** 10.3201/eid0604.000410

**Published:** 2000

**Authors:** O Lesens, Y Hansmann, P Riegel, R Heller, M Benaissa-Djellouli, M Martinot, H Petit, D Christmann

**Affiliations:** Service des Maladies Infectieuses et Tropicales, Clinique Médicale A, Hôpitaux Universitaires, Strasbourg, France. olivier.lesens@wanadoo.fr

## Abstract

We report the first case of endocarditis caused by a Gordonia species genetically related to G. sputi but exhibiting some atypical biochemical features in a 31-year-old woman with a central venous catheter. This unusual pathogen may be a new cause of opportunistic infections in patients with severe underlying diseases.


### Bacteremia and Endocarditis Caused by a *Gordonia* Species in a Patient with a Central Venous Catheter

**Olivier Lesens,* Yves Hansmann,* Philippe Riegel,† Rémy Heller,† Mohamed Benaissa-Djellouli,* Martin Martinot,* Hélène Petit,‡ Daniel Christmann,***

*Service des Maladies Infectieuses et Tropicales, Clinique Médicale A, Hôpitaux Universitaires, Strasbourg, France; †Laboratoire de Bactériologie, Faculté de Médecine, ‡Service de Chirurgie Cardio-Vasculaire, Hôpitaux Universitaires, Strasbourg, France

We report the first case of endocarditis caused by a *Gordonia* species genetically related to *G. sputi* but exhibiting some atypical biochemical features in a 31-year-old woman with a central venous catheter. This unusual pathogen may be a new cause of opportunistic infections in patients with severe underlying diseases.

*Gordonia* spp. are a gram-positive coryneform bacteria, recently identified in three patients as a cause of systemic infections (1,2), including two associated with indwelling implantable subcutaneous central venous catheters (2). We report *Gordonia* spp.'s capacity to infect a patient's undamaged cardiac valve through an implantable subcutaneous central catheter.

### Case Report

The patient was a 31-year-old woman, who had undergone splenectomy at age 9 years, for severe double heterozygous hemoglobinopathy (beta-thalassemia and hemoglobin E disease). Multiple blood transfusions at that time were complicated by hepatitis C with cirrhosis and secondary hemochromatosis, treated at home with twice weekly deferoxamine by subcutaneous central venous catheter. Hemochromatosis was complicated by diabetes, adrenal insufficiency, and peripheral neuropathy. In September 1997, the patient became ill with *Staphylococcus aureus* bacteremia associated with localized renal and cutaneous abscesses; transesophageal echocardiography showed neither valvular vegetation nor a valvular defect suggestive of endocarditis. The bacteremia was successfully treated with intravenous fosfomycin, cefotaxime and netilmicin, followed by ciprofloxacin and oxacillin for 8 weeks. The subcutaneous central venous port was also changed after 6 weeks of treatment.

The patient remained afebrile until December 1998, when fever and chills developed. Physical examination 1 week after onset of symptoms revealed a temperature of 39°C and a new mitral systolic murmur. The site of the subcutaneous central venous port showed no signs of infection. Results of clinical laboratory tests showed a leukocyte count of $31.4x10^9$/L, an erythrocyte sedimentation rate of 67 mm/h, and a C-reactive protein plasma level <5 mg/L. Transthoracic echocardiography revealed a mitral valvular vegetation (10x5 mm). Two blood cultures (one obtained from the central venous catheter) were performed at admission. One more peripheral blood sample was drawn for culture 12 hours later. All three cultures were positive for a gram-positive coryneform bacterium showing no extensive branching. This organism produced dry, raised, salmon-to-orange colonies without aerial hyphae. As the organism was weakly acid fast, according to the Kinyoun acid-fast stain modified for aerobic actinomycetes, we presumptively identified this organism as *Rhodococcus* sp. sensu lato. According to disk diffusion, the organism was susceptible to

Address for correspondence: Olivier Lesens, Service des Maladies Infectieuses et Tropicales, Clinique Médicale A, Fédération des Services de Médecine Interne, Hôpital Civil, 1 place de l'Hôpital - B.P. 426- 67091 Strasbourg Cedex, France; fax: 03 88 11 64 64; e-mail: olivier.lesens@wanadoo.fr.

penicillin G, amoxicillin, cefotaxime, ceftriaxone, imipenem, gentamicin, netilmicin, ciprofloxacin, vancomycin, and erythromycin but was resistant to fosfomycin, ceftazidime, trimethoprim-sulfamethoxazole, and streptogramin. By E-test, the MICs of penicillin G, amoxicillin, and ceftriaxone were 0.047, 0.064 and 0.25 µg/mL, respectively. The patient was successfully treated with intravenous amoxicillin (3 g, 4/day) and intravenous netilmicin (150 mg, 2/day) for 1 week, then with intravenous amoxicillin alone for 3 weeks. This treatment was followed by home treatment with 2 g perfusion of ceftriaxone for 2 weeks. The central venous catheter was left in situ. At 1 year follow-up, the patient was not infected with *Gordonia* sp., and echocardiographic findings were consistent with mitral valve insufficiency without oscillating intracardiac mass on valve.

To accurately identify the organism, we analyzed the p-bromophenacyl esters of mycolic acids of the isolate by using high-performance liquid chromatography, obtaining a pattern consistent with that of *Gordonia* sp. The number of peaks and retention times were similar to those exhibited by the *G. sputi* type strain ATCC 29627. Biochemical test results were positive for the hydrolysis of urease and esculin but negative for the hydrolysis of xanthine, adenine, tyrosine, and hypoxanthine. When inoculated with aerobic, low-peptone carbohydrate slants, the strain produced acid from trehalose but not from L-rhamnose and D-mannitol. These biochemical characteristics fit those of *Gordonia* species, especially *G. bronchialis* (3). To determine partial 16S rRNA gene sequence, the two eubacterial universal primers P8-27 (5'-AGA GTT TGA TCC TGG CTC AG-3') and P1392-1372 (5'-AAG GCC CGG GAA CGT ATT CAC-3') were used for the amplification, then a direct sequencing method with an internal primer P535-514 (5'-GTA TTA CCG CGG CTG CTG GGC AC-3') 5' labeled with fluorescein isothiocyanate was performed (4). The sequence obtained coincides with the 450 5' base pairs of the 16S rRNA gene and matches totally that of *G. sputi* present in the GenBank-EMBL database. This sequence did not match other bacterial sequences, including those of other *Gordonia* species. The sequence of the isolate differed from the sequence of the *G. aichiensis* type strain by two nucleotides. DNA–DNA similarity experiments, according to the stringent nuclease S1 method, showed 55% DNA relatedness with *G. sputi* type strain and less than 28% with the type strain of other species including *G. aichiensis.* (The genetic definition of a species is more than 70% DNA similarity.) Thus, this isolate does not fit in any of the recognized *Gordonia* species, although it is taxonomically close to *G. sputi*.

### Conclusions

The recent differentiation of *Gordonia* sp. as a distinct genus is the outcome of a taxonomic history complicated by several reclassifications (Table 1). Twelve organisms now belong to the genus *Gordonia*, including three species discovered in 1999 (5-9). To review the spectrum of clinical diseases in humans caused by *Gordonia* spp., we performed a Medline search for 1966 to 1999, using all the designations included in Table 1. Only *G. bronchialis* (10), *G. rubropertincta* (11),

Table 1. Classification of the genus *Gordonia*

| Present designated species (date) | Former designated species (date) |
|---|---|
| *Gordonia aichiensis*[a] (1997) | *Gordona aichiensis* (1994) ← *Rhodococcus aichiensis* (1983) |
| *Gordonia alkanivorans* (1999) | -- |
| *Gordonia amarae*[a] (1997) | *Gordona amarae* (1994) ← *Nocardia amarae* (1980) |
| *Gordonia bronchialis*[a] (1997) | *Gordona bronchialis* (1989) ← *Rhodococcus bronchialis* (1980) |
| *Gordonia desulfuricans* (1999) | -- |
| *Gordonia hirsuta*[a] (1997) | *Gordona hirsuta* (1996) |
| *Gordonia hydrophobica*[a] (1997) | *Gordona hydrophobica* (1995) |
| *Gordonia polyisoprenivorans* (1999) | -- |
| *Gordonia rhizosphera* (1998) | -- |
| *Gordonia rubropertincta*[a] (1997) | *Gordona rubropertincta* (1989) ← *Rhodococcus rubropertinctus*[b] (1980) |
| *Gordonia sputi*[a] (1997) | *Gordona sputi* (1989) ← *Rhodococcus sputi*[c] (1975) |
| *Gordonia terrae*[a] (1997) | *Gordona terrae* (1989) ← *Rhodococcus terrae* (1980) |

[a]The original spelling, *Gordona,* was changed to *Gordonia* in 1997 (5).
[b]Other former designations: *Bacillus rubropertinctus*, *Serratia rubropertincta*, *Mycobacterium rubropertinctum*, *Proactinomyces rubropertinctus*, *Nocardia rubropertincta.*
[c]Synonym: *Rhodococcus chubuensis.*

*G. sputi* (1,12), and *G. terrae* (2,13,14) have been shown to be pathogenic in humans (Table 2). They are derived from soil and may also be isolated in the sputa from patients with chest disorders (15). In an outbreak of sternal-wound infections (10), *G. bronchialis* was isolated from the hand, scalp, and vagina of a nurse as well as from her dog.

Systemic *Gordonia* spp. infections have been described in three patients. Buchman et al. (2) reported two cases of *Gordonia* spp. bloodstream infection associated with a Hickman catheter in two immunocompetent patients receiving long-term parenteral nutrition. Both strains were susceptible to vancomycin and gentamicin. In one case, the *Gordonia* sp. isolated from blood cultures and from a broth culture of the catheter tip was not clearly identified but was close to *G. rubropertincta*. The patient received intravenous vancomycin for 5 days and intravenous gentamicin for 19 days; the catheter was removed after 2 days. In the second case, the microorganism isolated from blood cultures was identified as *G. terrae,* and the patient was treated with intravenous vancomycin for 6 weeks with the catheter left in situ.

The only case of bacteremia caused by *G. sputi* was described in a 34-year-old immunocompromised patient (1) with metastatic melanoma treated with intravenous interleukin-2. The bacterium was thought to have reached the bloodstream through extensive desquamative skin rashes caused by the interleukin-2 treatment or by contamination of the catheter, although cultures from these specimens were negative. Because *G. sputi* was susceptible to ß-lactams, vancomycin, aminoglycosides, doxycycline, and rifampicin, the patient was first treated with amoxicillin-clavulanate (1,000 and 125 mg, respectively, every 3 hours). After 1 week of treatment, a second set of blood cultures yielded the same organism, and the treatment was changed to a combination of amikacin and piperacillin, which was successful (1). As with other *Gordonia* species, *G. sputi* may be isolated in the sputum of patients with pulmonary disease (15). Mediastinitis caused by *G. sputi* after coronary artery bypass grafting was recently described in an immunocompetent patient (12). The patient was treated with cefmetazole sodium (2 g per day for 3 weeks) and piperacillin sodium (2 g per day for 2 weeks) after surgical soft tissue debridement.

In our case, *Gordonia* sp. systemic infection associated with an implantable subcutaneous central venous catheter was complicated by endocarditis. The diagnosis was assessed as definitive on the basis of Duke criteria (one major and three minor, including echographic evidence of an oscillating intracardiac mass with a new regurgitant murmur, two persistently positive blood cultures yielding *Gordonia* sp., fever >38°C, and intravenous deferoxamin use). Our patient had neither neutropenia nor immunosuppressive medications, but underlying diseases may have impaired the immune system and facilitated infection. The bacteremia may be caused by manipulations of the implantable subcutaneous central venous catheter during routine home use. However, even if no other

Table 2. Types of infection caused by *Gordonia* species and patients' underlying conditions

| Type of infection | Cases (No.) | Age (yrs) | *Gordonia* species | Underlying conditions | Authors |
|---|---|---|---|---|---|
| Sternal wound | 7 | 51-68 | *G. bronchialis* | Surgery | Richet et al. (10) |
| Mediastinitis | 1 | 54 | *G. sputi* | Surgery | Kuwabara et al. (12) |
| Brain abscess | 1 | 40 | *G. terrae* | None | Drancourt et al. (14) |
| | 1 | 3 | *G. terrae* | Cerebral tumor | Drancourt et al. (13) |
| Lung infection | 1 | 29 | *G. rubropertincta* | Tuberculosis | Hart et al.(11) |
| Bacteremia due to central venous catheter | 2 | 43<br>65 | *Gordonia* sp[a]<br>*G. terrae* | Breast and ovarian cancer<br>Chronic intestinal pseudo-obstruction syndrome | Buchman et al. (2) |
| Bacteremia due to cutaneous lesions | 1 | 34 | *G. sputi* | Metastatic melanoma IL2 treatment[b] | Riegel et al. (1) |
| Skin infection | 1 | 7 | *G. terrae* | None | Martin et al. (16) |

[a]Not identified.
[b]IL2 = interleukin-2.

source for the bacteremia had been evident, no inflammation was observed at the port site, and semiquantitative culture was not available. An environmental investigation was not performed.

We conclude that *Gordonia* spp. may cause opportunistic infections, in particular bacteremia and endocarditis, in patients with severe underlying diseases and indwelling central catheters.

### Acknowledgment

The authors thank JP Euzéby (author of the folder "list of bacterial names with standing in nomenclature," http://www-sv.cict.fr/bacterio/) for providing helpful comments about *Gordonia*' s classification.

Dr. Lesens is a specialist in infectious diseases in the Department of Infectious Diseases, Strasbourg Hospital, France. He is pursuing a Ph.D. in epidemiology with scientific interests centered on the epidemiology of *Staphylococcus aureus* bacteremia.

### References

1. Riegel P, Ruimy R, de Briel D, Eichler F, Bergerat JP, Christen R, et al. Bacteremia due to *Gordona sputi* in an immunocompromised patient. J Clin Microbiol 1996;34:2045-7.
2. Buchman AL, McNeil MM, Brown JM, Lasker BA, Ament ME. Central venous catheter sepsis caused by unusual *Gordona* (*Rhodococcus*) species: identification with a digoxigenin-labeled rDNA probe. Clin Infect Dis 1992;15:694-7.
3. McNeil MM, Brown JM. The medically important aerobic *actinomycetes*: epidemiology and microbiology. Clin Microbiol Rev 1994;7:357-417.
4. Heller R, Jaulhac B, Charles P, de Briel D, Vincent V, Bohner C, et al. Identification of *Mycobacterium shimoidei* in a tuberculosis-like cavity by 16S ribosomal DNA direct sequencing. Eur J Clin Microbiol Infect Dis 1996;15:172-7.
5. Stackebrandt E, Rainey FA, Ward-Rainey NL. Proposal for a new hierarchic classification system, *Actinobacteria classis* nov. Int J Syst Bacteriol 1997;47:479-91.
6. Takeuchi M, Hatano K. *Gordonia rhizosphera* sp. nov. isolated from the mangrove rhizosphere. Int J Syst Bacteriol 1998;48:907-12.
7. Kummer C, Schumann P, Stackebrandt E. *Gordonia alkanivorans* sp. nov., isolated from tar-contaminated soil. Int J Syst Bacteriol 1999;49: 1513-22.
8. Kim SB, Brown R, Olfield C, Gilbert SC, Goodfellow M. *Gordonia desulfuricans* sp. nov. a benzothiophenedesulphurizing actinomycete. Int J Syst Bacteriol 1999;49: 1845-51.
9. Linos A, Steinbuchel A, Sproer C, Kroppenstedt RM. *Gordonia polyisoprenivorans* sp. nov., a rubber-degrading actinomycete isolated from an automobile tyre. Int J Syst Bacteriol 1999;49: 1785-91.
10. Richet HM, Craven PC, Brown JM, Lasker BA, Cox CD, McNeil MM, et al. A cluster of *Rhodococcus (Gordona) bronchialis* sternal-wound infections after coronary-artery bypass surgery. N Engl J Med 1991;324:104-9.
11. Hart DHL, Peel MM, Andrew JH, Burdon JGW. Lung infection caused by *Rhodococcus*. Aust N Z J Med 1988;18:790-1.
12. Kuwabara M, Onitsuka T, Nakamura K, Shimada M, Ohtaki S, Mikami Y. Mediastinitis due to Gordonia sputi after CABG. J Cardiovasc Surg (Torino) 1999;40:675-7.
13. Drancourt M, McNeil MM, Brown JM, Lasker BA, Maurin M, Choux M, et al. Brain abscess due to *Gordona terrae* in an immunocompromised child: case report and review of infections caused by G. terrae. Clin Infect Dis 1994;19:258-62.
14. Drancourt M, Pelletier J, Cherif AA, Raoult D. *Gordona terrae* central nervous system infection in an immunocompetent patient. J Clin Microbiol 1997; 35:379-82.
15. Tsukamura M. Proposal of a new genus, *Gordona*, for slightly acid-fast organisms occurring in sputa of patients with pulmonary disease and in soil. J Gen Microbiol 1973;25:665-81.
16. Martin T, Hogan DJ, Murphy F, Natyshak I, Ewan EP. *Rhodococcus* infection of the skin with lymphadenitis in a nonimmunocompromised girl. J Am Acad Dermatol 1991;24:328-32.

## References

[PDF_00284] Buchman A. L., McNeil M. M., Brown J. M., Lasker B. A., Ament M. E. (1992). Central venous catheter sepsis caused by unusual Gordona (Rhodococcus species: identification with a digoxigenin-labeled rDNA probe.. Clin Infect Dis.

[PDF_00325] Drancourt M., McNeil M. M., Brown J. M., Lasker B. A., Maurin M., Choux M., Raoult D. (1994). Brain abscess due to Gordona terrae in an immunocompromised child: case report and review of infections caused by G. terrae.. Clin Infect Dis.

[PDF_00330] Drancourt M., Pelletier J., Cherif A. A., Raoult D. (1997). Gordona terrae central nervous system infection in an immunocompetent patient.. J Clin Microbiol.

[PDF_00318] Hart D. H., Peel M. M., Andrew J. H., Burdon J. G. (1988). Lung infection caused by Rhodococcus.. Aust N Z J Med.

[PDF_00292] Heller R., Jaulhac B., Charles P., De Briel D., Vincent V., Bohner C., Piémont Y., Monteil H. (1996). Identification of Mycobacterium shimoidei in a tuberculosis-like cavity by 16S ribosomal DNA direct sequencing.. Eur J Clin Microbiol Infect Dis.

[PDF_00306] Kim S. B., Brown R., Oldfield C., Gilbert S. C., Goodfellow M. (1999). Gordonia desulfuricans sp. nov., a benzothiophene-desulphurizing actinomycete.. Int J Syst Bacteriol.

[PDF_00303] Kummer C., Schumann P., Stackebrandt E. (1999). Gordonia alkanivorans sp. nov., isolated from tar-contaminated soil.. Int J Syst Bacteriol.

[PDF_00321] Kuwabara M., Onitsuka T., Nakamura K., Shimada M., Ohtaki S., Mikami Y. (1999). Mediastinitis due to Gordona sputi after CABG.. J Cardiovasc Surg (Torino).

[PDF_00310] Linos A., Steinbüchel A., Spröer C., Kroppenstedt R. M. (1999). Gordonia polyisoprenivorans sp. nov., a rubber-degrading actinomycete isolated from an automobile tyre.. Int J Syst Bacteriol.

[PDF_00338] Martin T., Hogan D. J., Murphy F., Natyshak I., Ewan E. P. (1991). Rhodococcus infection of the skin with lymphadenitis in a nonimmunocompromised girl.. J Am Acad Dermatol.

[PDF_00289] McNeil M. M., Brown J. M. (1994). The medically important aerobic actinomycetes: epidemiology and microbiology.. Clin Microbiol Rev.

[PDF_00314] Richet H. M., Craven P. C., Brown J. M., Lasker B. A., Cox C. D., McNeil M. M., Tice A. D., Jarvis W. R., Tablan O. C. (1991). A cluster of Rhodococcus (Gordona) Bronchialis sternal-wound infections after coronary-artery bypass surgery.. N Engl J Med.

[PDF_00280] Riegel P., Ruimy R., de Briel D., Eichler F., Bergerat J. P., Christen R., Monteil H. (1996). Bacteremia due to Gordona sputi in an immunocompromised patient.. J Clin Microbiol.

[PDF_00300] Takeuchi M., Hatano K. (1998). Gordonia rhizosphera sp. nov. isolated from the mangrove rhizosphere.. Int J Syst Bacteriol.

